# Society Meetings

**Published:** 1902-08

**Authors:** 


### SOCIETY MEETINGS.

The American Association of Obstetricians and Gynecologists will hold its fifteenth annual meeting in the Convention Hall of the Hotel Raleigh, Washington, D.C., Tuesday, Wednesday and Thursday, September 16, 17 and 18, 1902, under the presidency of Dr. Edwin Ricketts, of Cincinnati. The manager of the Raleigh, Mr. T. J. Talty, should be addressed in relation to rooms; the secretary, Dr. William Warren Potter, 284 Franklin Street, Buffalo, should be communicated with on all other subjects pertaining to the meeting.

All physicians, and especially those in Washington and vicinity, are most cordially invited to the scientific sessions.

SCIENTIFIC PROGRAM.

The following-named papers have been offered:

1. President's address, Edwin Ricketts, Cincinnati.

2. Normal saline solutions during abdominal operations, Wm. H. Humiston, Cleveland.

3. Further notes on ovarian transplantation, Robert T. Morris, New York.

4. Surgery of the ileo-cecal valve, N. Stone Scott, Cleveland.

5. Ice following abdominal sections, Frank F. Simpson, Pittsburg.

6. Etiology and prophylaxis of traumatism of the female pelvic tract following labor, E. J. Ill, Newark.

7. Pelvic disease in the unmarried, C. L. Bonifield, Cincinnati.

8. Report of a few cases of operation for peritoneal tuberculosis with remarks, Rufus B. Hall, Cincinnati.

9. Suppression of urine as a post-operative condition, T. J. Crofford, Memphis.

10. Occipito-posterior positions, J. M. Duff, Pittsburg.

11. Intestinal anastomosis, J. A. Lyons, Chicago.

12. Retained placenta due to uterine fibroids, with cases, M. A. Tate, Cincinnati.

13. Hydramnion with report of a case, E. F. Fish, Milwaukee.

14. (*a*) Personal experiences in the use of the angiotribe; (*b*) Two fatal cases of tetanus, following abdominal section, Walter B. Dorsett, St. Louis.

15. A second contribution to the treatment of gastric ulcer, Henry Howitt, Guelph.

16. Hemorrhagic cysto-sarcomata of the ovary; their infectious character, J. B. Murphy, Chicago.

17. General consideration of drainage in abdominal and pelvic surgery, Joseph Price, Philadelphia.

18. Surgical relations that the appendix region or zone bear to pelvic suppuration and operative complications, Joseph Price, Philadelphia.

19. Infantile intestinal diverticula, Joel W. Hyde, Brooklyn.

20. Myomectomy vs. hysterectomy, C. C. Frederick, Buffalo.

21. Anterior transplantation of the round ligaments for uterine displacements, A. H. Ferguson, Chicago.

22. The cases of peritonitis which recover after operation, Wm. E. B. Davis, Birmingham.

23. Dystocia following ventrofixation of the uterus, William H. Wenning, Cincinnati.

24. Surgery of the ureters, C. R. Dudley, St. Louis.

25. Curetage in streptococcus infection of the uterus, D. Tod Gilliam, Columbus.

26. A new operation for displaced kidney with report of cases, Charles A. L. Reed, Cincinnati.

27. Cancer of the large intestines with special reference to Mikulicz's method of resection, M. Stamm, Fremont, O.

28. A short review of the advances made in intestinal surgery, James F. W. Ross, Toronto.

29. Ectopic pregnancy, H. D. Ingraham, Buffalo.

30. Unusual cases of appendicitis, Miles F. Porter, Fort Wayne.

31. Pelvic abscess and its treatment, H. E. Hayd, Buffalo.

32. Abdominal section during pregnancy, J. H. Carstens, Detroit.

33. Ruptured pus tubes, Charles G. Cumston, Boston.

34. Title to be announced, H. O. Pantzer, Indianapolis.

35. Gastrectomy with report of two cases, A. Vander Veer, Albany.

36. Four cases illustrating the difficulties of diagnosticating appendicitis, William Wotkyns Seymour, Troy.

37. The vaginal route for operations on the uterus and appendages, with cases, Jos. H. Branham, Baltimore.

38. The management of cases of emergency arising from rupture in ectopic pregnancy, A. P. Clarke, Cambridge.

39. Some unsettled questions in the surgery of the appendix, John Young Brown, Jr., St. Louis.

40. Deciduoma malignum, with report of a case, L. S. McMurtry, Louisville.

41. Relationship of the colon to abdominal tumors, James F. Baldwin, Columbus.

42. Some problems in exploratory laparatomy, Walter B. Chase, New York.

43. Title to be announced, L. H. Dunning, Indianapolis.

44. Extirpation of the gall-bladder, through the lumbar incision; with report of a case, W. P. Manton, Detroit.

---

The Medical Society of the County of Chautauqua held its annual meeting at the Hotel Atheneum, Chautauqua, N. Y., Tuesday, July 8, 1902. The following program, prepared by the secretary, Dr. Charles A. Ellis, was observed: President's address, N. E. Beardsley, Dunkirk; Some ocular symptoms in general diseases, Geo. E. Blackham, Dunkirk; Presentation of cases by Wm. Seaman Bainbridge, New York: (1) fracture of the neck of the humerus; (2) fibromyomata uteri—hysterectomy; (3) fibromyomata uteri—myomectomy; (4) prostatic hypertrophy—patient aged 75 years, operation, cured; Immunity, Henry R. Hopkins, president of the Medical Society of the State of New York, Buffalo.

---

The New York State Medical Association—fourth district branch—held its eighteenth annual meeting at Hotel Atheneum, Chautauqua, N. Y., July 23. The following was the program: President's address, Chas. A. Wall, Buffalo; Congenital narrowness of the aortic system, Joseph Burke, Buffalo—discussion, Chas G. Stockton, DeLancey Rochester, Buffalo; The broad therapeutic application of ergot, Alfred T. Livingston, Jamestown—discussion, H. D. Ingraham, Buffalo; The differential diagnosis of diseases of the hip-joint, Wisner R. Townsend, New York—discussion, Wm. C. Phelps, Buffalo, Wm. M. Bemus, Jamestown; Fecal fistula, Frederick Holme Wiggin, New York—discussion, Eugene A. Smith, Buffalo, Marcell Hartwig, Buffalo; Eczema,

Grover W. Wende, Buffalo—discussion, Alfred E. Diehl, Buffalo.

---

The Fourteenth International Congress of Medicine will be held in Madrid, Spain, from April 23 to 30, 1903, under the patronage of their majesties, the King of Spain and the Queen-mother. The president of the congress is Professor Julian Celleja y Sanchez, the general secretary is Dr. Angel Fernandez-Caro, and the general treasurer is Professor José Cómez Ocaña. The preliminary statement of regulations and program has been issued, and it announces that members of the congress will be physicians, pharmacists, dentists, veterinary surgeons, and other persons working in branches of medical science, both Spaniards and foreigners, who have entered their names and paid their subscriptions. Other persons who possess scientific or professional titles, and who wish to take part in the work of the congress, may share in it under the above conditions. Communications should be accompanied by a short abstract, which will be printed and distributed among the members of the congress and should be addressed to J. H. Huddleston, secretary, American committee, 126 West 85th Street, New York City.

---

The American Electo-therapeutic Association will hold its annual meeting at the Hotel Kaaterskill, Catskill Mountains, New York, September 2, 3 and 4, 1902.

---

The National Association of United States Pension Examining Surgeons was organised at Saratoga Springs, June 9, 1902.

The meeting at Saratoga was successful, a large number of enthusiastic examining surgeons were present, and officers were elected for the coming year. Several interesting papers were presented and by special invitation, Dr. J. F. Raub, Medical Referee of the U. S. Pension Bureau, read a paper full of valuable suggestions concerning the work of all pension examining surgeons. This, by vote of the association, is to be printed and sent to examining surgeons throughout the country.

During the coming year a vigorous and earnest attempt is to be made to interest every pension examining surgeon in the United States in this organisation, and to induce as many as possible to join it. Inasmuch as these number about 4,500 physicians, it is evident that the association is probably destined to become an important factor among medical societies in the United States.

The officers elected for the ensuing year are: president, Wm. A. Howe, Phelps, N.Y.; vice-presidents, Wm. H. Hall, Saratoga Springs, N.Y., Cyrus L. Stevens, Athens, Pa., Charles James Fox, Willimantic, Conn., G. Law, Greeley, Col.; secretary, Wheelock Rider, Rochester, N.Y.; treasurer, Charles H. Glidden, Little Falls, N.Y.

The executive committee is made up as follows: The president, *ex-officio*; F. W. Firmin, Findlay, O.; John VanRensselaer, Washington, D.C.; J. Sutcliffe Hill, Bellows Falls, Vt.; Warren E. Anderson, Pensacola, Fla.; Henry Allers, Newark, N.J.; J. H. Maxwell, Newton, Ill.; G. Lane Taneyhill, Baltimore, Md.; and Joseph E. Jones, Desoto, Mo.

All members of pension examining boards and all expert examiners are eligible for membership, and any such may become a member by sending his name and the dues for one year ($1.00) to the treasurer, Charles H. Glidden, M.D., Little Falls, N.Y.

---

The Buffalo Academy of Medicine.—All members are invited and urged to contribute to the program for the session of 1902-1903, and to communicate with the officers, as announced below: Section of medicine—chairman, A. E. Woehnert; secretary, Julius Ullman, 400 Franklin Street. Section of surgery: chairman, Henry Rooth; secretary, Marshall Clinton, 466 Franklin Street. Section of obstetrics: chairman, L. Schroeter; secretary, Henry Stadlinger, 274 Swan Street. Section of pathology: chairman, Irving P. Lyon; secretary, Jacob S. Otto, 394 Franklin Street. Section of ophthalmology: chairman, E. E. Blaauw; secretary, F. H. Millener, 724 Main Street. The council would appreciate the assistance of members in securing the services of out-of-town men of note in the profession to participate in the program. Herman E. Hayd, president; F. W. McGuire, 1540 Main Street, secretary.

---

The Buffalo Academy of Medicine, section of ophthalmology, otology, laryngology and rhinology has recently issued the following special circular:

Sufficient interest in this section has not been evidenced in the past by the support extended to it by members of the academy, and it was, perhaps, due to a misconception that the meetings would only be of interest to those who devote themselves to these branches. Therefore, in order that the meetings of this section, for the coming year, may be made highly successful, we ask you to send us the title of some subject, falling within the limits of the section, in which you are particularly interested. It is very important that we should have the sympathy and active support of every member of the academy. Your kind attention and an early reply, will be much appreciated. (Signed) Edmund E. Blaauw, chairman; George F. Cott, secretary.

